# Vaccine effectiveness of inactivated and mRNA COVID-19 vaccine platform during Delta and Omicron wave in Jakarta, Indonesia: A test-negative case-control study

**DOI:** 10.1371/journal.pone.0320779

**Published:** 2025-06-09

**Authors:** Erlina Burhan, Farchan Azzumar, Fira Alyssa Gabriella Sinuraya, Sabarinah Prasetyo, Dwi Gayatri, Iwan Ariawan, Muhammad Ilham Dhiya Rakasiwi, Hanna Lianti Afladhia, Ahmad Fadhil Ilham, Ihya Akbar, Elvan Wiyarta

**Affiliations:** 1 Indonesia Society of Respirology, Jakarta, Indonesia; 2 Department of Pulmonology and Respiratory Medicine, Faculty of Medicine Universitas Indonesia, Jakarta, Indonesia; 3 Persahabatan National Respiratory Referral Hospital, Jakarta, Indonesia; 4 Respiratory Programmatic Implementation and Research Institute, Jakarta, Indonesia; 5 Faculty of Public Health Universitas Indonesia, West Java, Indonesia; SRM Medical College Hospital and Research Centre, INDIA

## Abstract

**Background:**

Vaccination was included in the Indonesian government policy to address Delta and Omicron waves of SAR-CoV-2 infections. This study assesses the effectiveness of inactivated (CoronaVac, BBIBP-Cor) and mRNA vaccines (mRNA-1273, BNT162b2) against COVID-19 regardless of symptoms and fatal COVID-19 (mortality within 30 days after confirmed RT-PCR) during Delta and Omicron period in Jakarta, Indonesia.

**Methods:**

This study case-control, test-negative study included all individuals aged over 18 years in Jakarta with complete and consistent SARS-CoV-2 RT-PCR results from 1 June to 31 August 2021 (Delta period) and 1 January to 2 April 2022 (Omicron period), as well as complete vaccination status. This study integrates several public health data from the Jakarta provincial government. From the odds ratio, vaccine effectiveness (VE) was analyzed as the primary outcome using unmatched analysis, matched analysis, and adjustments for other factors.

**Results:**

This study includes 982,885 eligible subjects recorded from March 2021 to April 2022. All subjects generally underwent testing 4–9 weeks after their last vaccine dose. The VE of 2-dose inactivated vaccine against SARS-CoV-2 infection during Delta wave was 22.06% (95% CI 20.63–24.54) and the VE against fatal COVID-19 was 78.55% (95% CI 72.91–83.00). A complete primary dose of mRNA vaccine showed VE of 24.81% (95% CI 16.81–32.09) against infection during Omicron wave. Furthermore an additional mRNA booster dose showed VE of 68.82% (95% CI 54.11–78.82) based on unmatched analysis.

**Conclusion:**

A complete primary dose of inactivated vaccine provided mild protection against COVID-19 and essential protection against fatal cases during the Delta wave, but offered little to no protection during the Omicron wave. In contrast, the mRNA vaccine, either as primary vaccination, homologous, or heterologous booster regimen, conferred acceptable protection against Omicron. This study recommends real-world vaccination strategies for LMICs with typical vaccine supply constraints.

## Introduction

Vaccination is one of the most effective global health interventions that can reduce mortality and morbidity or even eradicate vaccine-preventable diseases [1]. From the first year of the vaccination campaign, global COVID-19 vaccination had changed the trajectory of the pandemic and was expected to prevent 63% of total excess deaths between December 2020 to December 2021 [2]. Meta-analysis studies had confirmed that COVID-19 vaccine could prevent fatal, severe, and even asymptomatic infections [3–5]. However, most published results came from developed countries, while 80% of the global population resided in low- and middle-income countries (LMICs) [6,7]. These developing countries might have a different demographic and access to COVID-19 vaccines, thus implementing vaccination policies that differ from those in developed countries [8,9]. Quantifying the impact of these vaccination strategies on vaccine performance is an essential lesson learned for future national vaccination programs during public health crises [10].

As a fruitful marathon diplomatic effort of vaccine cooperation, Indonesia commenced its national COVID-19 vaccine rollout on 13 January 2021, 35 days after the United Kingdom (UK) started its national COVID-19 vaccine program. Among the 11 approved COVID-19 vaccines, CoronaVac (Sinovac) and BBIBP-CorV (Sinopharm) were the first vaccines that were included in Indonesian national COVID-19 vaccine rollout for two-dose series of primary regimen and were approved by Badan Pengawas Obat dan Makanan (BPOM), the Indonesian Regulatory Authority for Food and Drugs Administration [10]. Both CoronaVac (Sinovac) and BBIBP-CorV (Sinopharm) are inactivated vaccines, while Sinovac utilized the CN2 virus strain, the Sinopharm used the HB02 virus strains.

As an effort to mitigate the spread of the SARS-CoV-2 variant of concern (VOC) Omicron, the Ministry of Health (MoH) issued a recommendation to administer a booster vaccine six months after the two-dose primary vaccination, with ChAdOx1 nCoV-19 (AstraZeneca), BNT162b2 (Pfizer) and mRNA-1273 (Moderna) vaccine as the first booster regimen option for the national vaccination program on 12 January 2022. However, there was a subsequent revision on February 25, 2022, urging an expedited administration of the booster, into three months after the two-dose primary regimen [11].

The unmet gap between methodological challenges and data availability is an obstacle that researchers in resource-limited countries should overcome [12]. Test-negative case-control design is considered the best option for vaccine effectiveness study in LMICs because this approach is an accurate method with beneficial efficiency in terms of cost and time compared to cohort study [13].

This study is part of the RECOGNISE (Real-world effectiveness of CoronaVac, BBIBP-CorV, and mRNA-1273 in Jakarta) research group. It aims to evaluate the VE of CoronaVac (Sinovac), BBIBP-CorV (Sinopharm), mRNA-1273 (Moderna), and BNT162b2 (Pfizer) against SARS-CoV-2 infection (COVID-19) during a case surge associated with Delta and Omicron waves of the SARS-CoV-2 variant in Jakarta, Indonesia.

## Methods

### Population and study design

This RECOGNISE study used a case-control test-negative design (TND), a study design frequently used to estimate vaccine effectiveness against seasonal influenza due to its tangible advantages in both logistical ease and minimization of several biases [14,15]. In this study, we used a test-negative design by classifying the subjects with positive results of SARS-CoV-2 into cases, while the subjects with positive results of SARS-CoV-2 are classified as controls regardless of vaccination status to address three potential biases. First, cases and controls seek care at the same facilities, reducing bias from community-level differences in vaccine access and disease risk. Second, since they are tested for similar indications, it minimizes bias from variations in healthcare-seeking behavior, a common issue in traditional case-control studies. Lastly, vaccine status is recorded at or even before specimen collection, before test results are known, lowering the risk of misclassification [13].

The major goal of this study is to assess VE against SARS-CoV-2 infection regardless of symptoms during the study period and estimate the VE against fatal COVID-19 defined as mortality within 30 days after being confirmed by the first positive results of RT-PCR SARS-CoV-2 test. This study developed both unmatched and matched case-control datasets for the analysis. The matching procedure was done for each case with a ratio of 1:1 or 1: 2 by matching factors of 10-year age range, gender, and calendar week of index sample collection. Matching based on these factors has been shown to effectively control for differences in SARS-CoV-2 exposure risk in VE studies using TND [16,17].

We conducted a feasibility study to assess several datasets provided by the Jakarta Health Provincial Health Office and then determined the datasets required to address research questions. We found that New All Record (NAR) datasets, the national registry for COVID-19 testing that was mostly based on RT-PCR, serve as the foundation for case and control identification, and Primary Care Vaksin (PCare Vaksin) datasets, the national registry for COVID-19 registration, serve as the foundation for identifying vaccination status. These two datasets are combined and built into multiple sets for analysis.

We employed the total sampling method by collecting data from the entire population of Jakarta. Following the identification of SARS-CoV-2 findings throughout the study period (1 June-31 August 2021 for Delta variant-related wave and 1 January-2 April 2022 for Omicron), all participants were screened based on eligibility criteria in collaboration with datasets integration. Although most subjects had more than one RT-PCR result (negative, positive, or both) at different time points throughout the study period, this study only accounted for one sample with a date specific per subject (index sample) to determine this subject's assignment in case or control group. To ensure data completeness for each subject, verification was performed by cross-referencing the PCare Vaksin records with other datasets using the national ID numbers.

The study included male and female subjects aged at least 18 years, residing in Jakarta, with complete and consistent SARS-CoV-2 RT-PCR results, and complete and consistent vaccination status; for those who were vaccinated, the delta subset analysis study only included those who received inactivated vaccine because mRNA vaccine was not available for primary series vaccine campaign during the delta wave. Meanwhile, for the omicron subgroup analysis, the study evaluates inactivated and mRNA as primary series and as booster regimen regardless of the primary vaccine. In this study, planned eligibility criteria were modified to include both symptomatic and asymptomatic patients to have VE against SARS-CoV-2 infection instead of VE against symptomatic COVID-19. This action was dedicated to control information bias due to symptom status misclassification in source data and to control selection bias by expanding eligible subjects. The inappropriate recording of symptom status was caused by inaccuracies in documenting the initial date of symptom onset, either due to patient recall bias or the different data collection methods across the datasets. Data quality frailties due to format and variable inconsistencies during the COVID-19 pandemic have been reported worldwide, causing a significant number of missing data [18–22].

The study then dropped subjects who had a positive RT-PCR test result within 90 days of the index sample to avoid misclassification due to prolonged RT-PCR positivity, heterologous primary vaccine combination, 2-dose and 3-dose vaccine outside the recommended interval. Additionally, we excluded subjects who had an interval of <14 days after the primary dose or a gap of <7 days between the booster dose and index sample. Given the possible contribution of past infection to COVID-19 protection, previous infection was defined as an RT-PCR SARS-CoV-2 positive test result that occurred more than 90 days before the period of interest.

### Outcome and vaccination status

The primary outcome of this study is to evaluate the VE against SARS-CoV-2 infection. The secondary outcomes of the study are to evaluate (1) VE against mortality after 30 days of SARS-CoV-2 positive result, (2) VE against SARS-CoV-2 infection based on prior history of COVID-19 infection, and (3) VE against SARS-CoV-2 infection changes over time. The assessment of VE was divided based on the dominant SARS-CoV-2 variants in Jakarta: Delta (June 1 - August 31, 2021) and Omicron (January 1 - April 2, 2022).

Eligible individuals are assigned to either the case or control group based on the RT-PCR results of their initial sample. The initial sample is defined as the first sample with a positive RT-PCR result for those in the case group and the first sample with a negative result for those in the control group within the study period. Participants in the case group had at least one positive SARS-CoV-2 RT-PCR result during the study, whereas those in the control group had only negative results throughout the study period. To assess vaccination status during the relevant time frames, each eligible participant was matched to the vaccination dataset using a national unique 16-digit identity number. This allowed verification of their vaccination status (unvaccinated, one dose, or two doses), type of vaccine administered, and the vaccination date.

### Statistical analysis

The results of descriptive analyses were provided using mean and standard deviation (SD) or median and interquartile range (IQR) as applicable. The estimated VE was determined using the WHO recommendation formula [VE = (1-aOR) 100%] [23]. Those who received 2-dose and 1-dose vaccinations will be compared against those who were not vaccinated during the corresponding period of interest to obtain VE against infection, respectively. To see mRNA booster VE across different primary vaccination types, stratification was also performed for Omicron subset analysis. To see VE changes over time, we analyzed only those vaccinated (3-dose, 2-dose, or 1-dose) in a specific time-since-vaccination stratum and those unvaccinated as our reference group.

The matching procedure was assessed with standardized mean difference (SMD), with a value of less than 0.1 indicating sufficient matching [24]. The matched model was further adjusted for domicile (Central Jakarta, West Jakarta, North Jakarta, East Jakarta, South Jakarta, Kepulauan Seribu Regency), occupation (public, health worker, government staff), and history of previous COVID-19.

Unmatched analysis for VE against fatal cases within 30 days of the index sample date in Delta Wave (fatal COVID-19) was performed using multivariate analysis, adjusting for age (45 years and 45 years), domicile (Central Jakarta, West Jakarta, North Jakarta, East Jakarta, South Jakarta, Kepulauan Seribu Regency), occupation (public, health worker, government staff), week of PCR test, and history of previous COVID-19 (>3 months before the index PCR test). Unmatched multivariable logistic regression was carried out, especially when the conditional model failed to converge.

A two-sided *p-*value derived from logistic regression analysis was used to compare effectiveness. Variables that act as confounders were included, while those that act as colinear were excluded. To ensure correct linear function, continuous variables were transformed into categorical variables based on their relationship with the log odds of outcome. Model building was carried out using forward inclusion, and model fit was assessed by the Wald test and observing the changes in the primary exposure's beta coefficient standard error. Overall goodness-of-fit and multicollinearity of the final logistic model were made with the likelihood ratio test and variance inflation factor (VIF), respectively. The 95% confidence intervals (CIs) did not factor for multiplicity, and interactions were not examined [25,26]. Google Collaboratory® with Python® language programming was utilized for data integration and construction, while statsmodels [27] was used for estimation of statistical models and analysis.

### Ethics approval and consent

Before conducting the study, investigators declare that ethical approval has been obtained from the Persahabatan Hospital Health Research Ethics Committee by submitting the research protocol and all documents required (40/KEPK-RSUPP/05/2022). The Health Research Ethics Committee of Persahabatan Hospital has also declared an informed consent waiver for this study (DP.04.03/D.XX.10.4/0001/2024). This research has received a research permit from the Jakarta Provincial Health Office (2105/SDK/VII/2022). To ensure the security of research data and the privacy of the subjects, we did not collect subject names and de-identified their national ID numbers. The entire datasets are stored in a cloud system that is accessible only to the research team and the Jakarta Health Department, the data owner. The data is not shared with any external parties without explicit permission from the Jakarta Health Department. Access to the data for contributors has been revoked following the data collection process.

## Results

**Fig 1:**
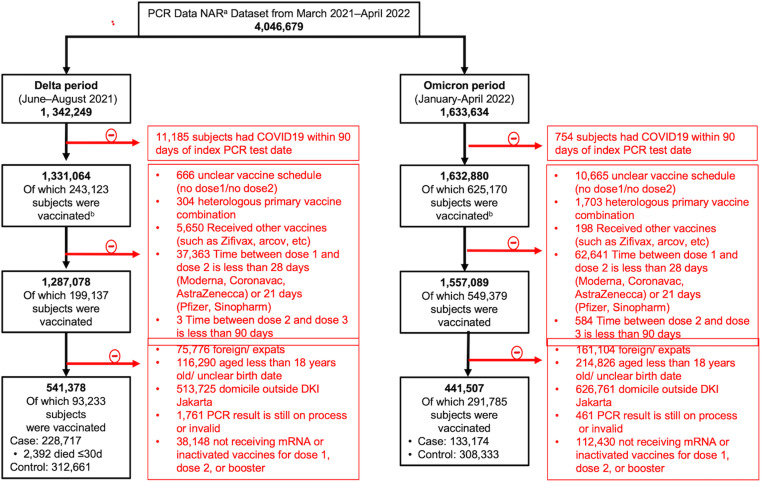
Data integration and eligibility assessment flow for delta and omicron subset analysis. *Matching according to gender, 10-year age range, calendar week of PCR test for the booster vaccine efficacy analysis. **(a)** New all record (NAR): consisted of information such as national ID number, gender, date of birth, domicile, date of PCR test, and result of PCR test. **(b)** Merged with vaccination dataset from the community health center and ID registry: consisted of information such as national ID number, gender, date of birth, domicile, date of vaccination, dose status, and name of the vaccine.

From the backbone dataset (4,046,679 subjects), there were 1,342,249 subjects having RT-PCR results in the Delta wave period and 1,633,634 subjects allocated in the Omicron period. After assessing eligibility in corresponding periods of interest, 541,378 and 441,507 subjects were eligible for unmatched analysis in Delta and Omicron subset analysis, respectively (Fig 1). The matching process wasdone both in Delta and Omicron subset analysis indicated by all matching factors’ SMD < 0.1 yielding 1:1 ratio for both 2-dose and 1-dose in Delta set analysis and 1:2 ratio for both 2-dose and booster in Omicron set analysis. A matching procedure for the secondary objective (VE against fatal COVID-19 and VE by time after vaccination) was not conducted due to an insufficient number of matching pairs obtained.

### Sociodemographic characteristics

**Fig 2:**
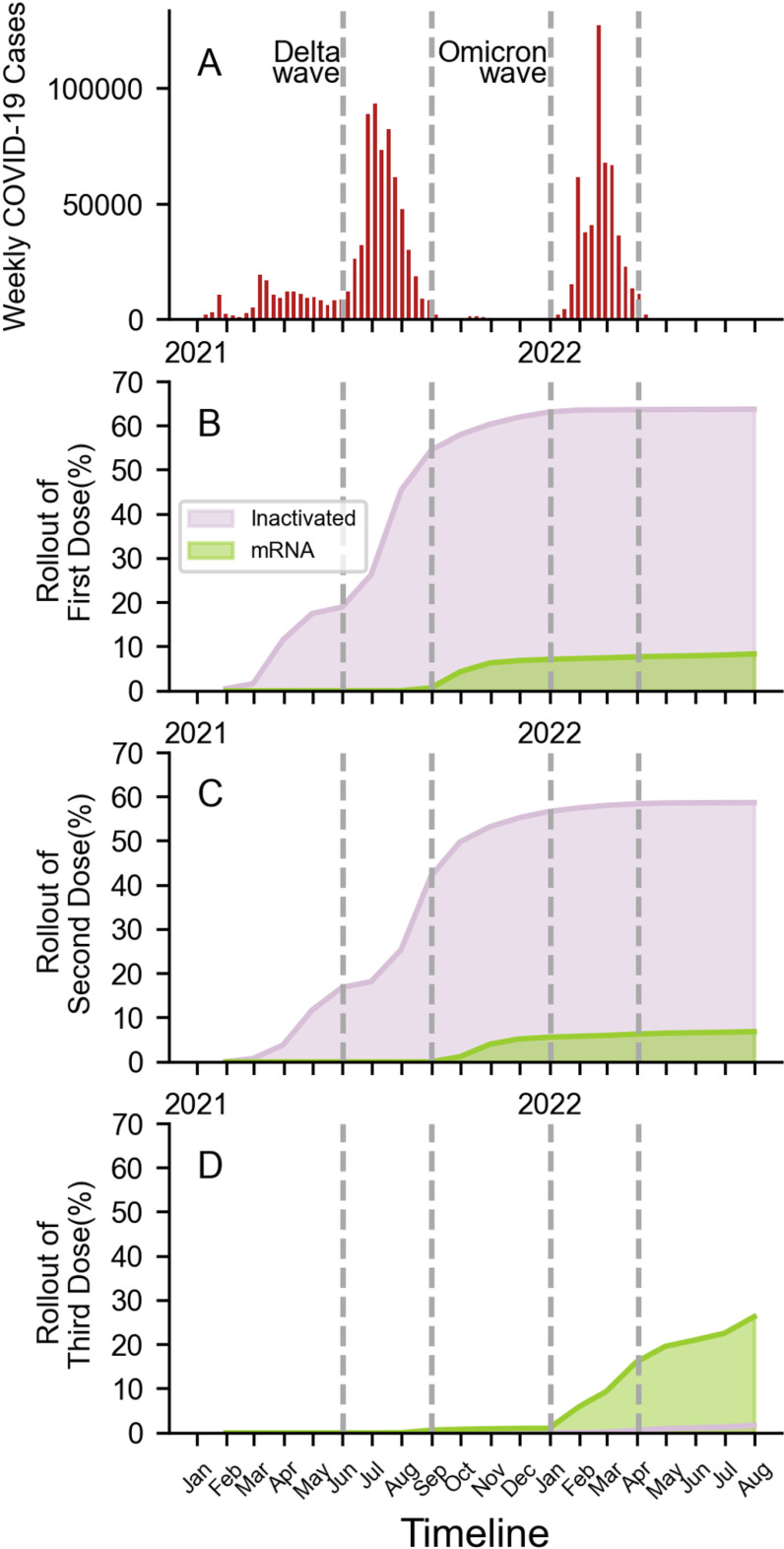
Cumulative case of COVID-19 and vaccination roll out in Jakarta. **(A)** Weekly number of COVID-19 cases during the epidemic waves caused by the Delta and Omicron variants in Indonesia; Cumulative proportion of the population who received a first **(B)**, second **(C)**, and third or booster dose (D) of each vaccine.

Our findings showed that the respondents’ age and sex in both the case and control groups were relatively comparable, with ages ranging from 37 to 39 years and male respondents constituting 46% to 48%, respectively (Table 1). Subjects hailed from all areas of Jakarta, evenly distributed according to the population profile of each region (Central Jakarta, West Jakarta, North Jakarta, East Jakarta, South Jakarta, Kepulauan Seribu Regency). There were more subjects with documented previous COVID-19 during the Omicron period compared to the Delta period (Fig 2).

**Table 1:** Sociodemographic characteristics of cases and control group during COVID-19 Delta and Omicron wave in Jakarta, Indonesia.

Characteristic	Delta wave (1 June–31 August 2021)			Omicron wave (1 January–2 April 2022)	
	Cases PCR+ N=228,717 (%)	Death ≤30 days among PCR+ N= 2,392 (%)	Controls PCR- N=312,661 (%)	Cases PCR+ N=133,174 (%)	Controls PCR- N=308,333 (%)
**Median age, years (Q1–Q3)**	39 (28–52)	58 (50–68)	37 (28–50)	37 (28–51)	37 (28–51)
**Male gender**	109,468 (47.86)	1,257 (52.55)	150,537 (48.15)	61,369 (46,08)	148,922 (48,30)
**Occupation**					
General public	194,440 (85.01)	2,353 (98.37)	252,532 (80.77)	108,569 (81.52)	249,398 (80.89)
Government sector	27,913 (12.20)	32 (1.34)	48,295 (15.45)	21,968 (16.50)	53,712 (17.42)
Health sector	6,364(2.78)	7 (0.29)	11,834 (3.78)	2,637 (1.98)	5,223 (1.69)
**Domicile**					
West Jakarta	48,247 (21.09)	492 (20.57)	63,681 (20.37)	30,368 (22.80)	68,346 (22.17)
South Jakarta	54,099 (23.65)	521 (21.78)	76,337 (24.42)	34,156 (25.65)	80,420 (26.08)
East Jakarta	69,097 (30.21)	778 (32.53)	84,163 (26.92)	33,702 (25.31)	73,631 (23.88)
North Jakarta	32,202 (14.08)	317 (13.25)	50,861 (16.27)	19,270 (14.47)	47,519 (15.41)
Central Jakarta	24,548 (10.73)	281 (11.75)	36,860 (11.79)	15,579 (11.70)	38,155 (12.37)
Kepulauan Seribu Regency	524 (0.23)	3 (0.13)	759 (0.24)	99 (0.07)	262 (0.09)
**Median week of PCR test (Q1–Q3)**	6 (4–7)	7 (4–8)	7 (4–9)	8 (6–9)	7 (4–9)
**Previous COVID-19 infection**	553 (0.24)	5 (0.21)	3,927 (1.26)	8,274 (6.21)	25,395 (8.24)
**Vaccination status (at the time of PCR test)**					
Unvaccinated	201,351 (88.04)	2,228 (93.14)	246,794 (78.93)	44,038 (33.07)	105,684 (34.28)
Dose 1	7,334 (3.21)	40 (1.67)	16,794 (5.37)	2,558 (1.92)	8,648 (2.80)
mRNA vaccine	7 (0.10)	0 (0.00)	109 (0.65)	543 (21.23)	2,081 (24.06)
Inactivated vaccine	7,327 (99.90)	40 (0.00)	16,683 (99.35)	2,015 (78.77)	6,567 (75.94)
Dose 2	20,029 (8.76)	124 (5.18)	48,993 (15.67)	70,442 (52.89)	154,747 (50.19)
mRNA vaccine	1 (0.01)	0 (0.00)	2 (0.00)	5,782 (8.21)	16,100 (10.40)
Inactivated vaccine	20,028 (99.99)	124 (100.00)	48,991(100.00)	64,660 (91.79)	138,647 (89.60)
Booster	3 (0.00)	0 (0.00)	80 (0.03)	16,136 (12.12)	39,254 (12.73)
mRNA booster	3 (100.00)	NA	79 (98.75)	15,560 (96.43)	38,154 (97.20)
Inactivated primary vaccine	3 (100.00)	NA	79 (98.75)	14,338 (92.15)	35,090 (91.97)
ChAdOX1 nCoV-19 primary vaccine	NA	NA	NA	1,179 (7.58)	2,796 (7.33)
**Time since last vaccination (at the time of PCR test)**					
Median days since last dose 1 (Q1–Q3)	21 (12–36)	25 (14–92)	21 (11–34)	159 (90–214)	147 (78–209)
Median days since last dose 2 (Q1–Q3)	63 (41–84)	77 (58–107)	59 (26–93)	197 (168–259)	195 (154–255)
Median days since last booster (Q1–Q3)	6 (3–8)	NA	7 (3–15)	23 (11–39)	24 (11–40)

### Protection by inactivated vaccine

**Fig 3:**
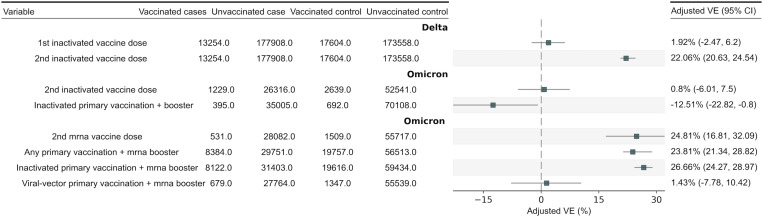
Forest plot for the matched analysis on VE against SARS-CoV-2 infection during Delta and Omicron wave. *Adjusted VE means case and controls were matched exactly in a 1:1 or 1:2 ratio according to gender, 10-year age range, and calendar week of PCR test. Further adjusted to domicile (Central Jakarta, West Jakarta, North Jakarta, East Jakarta, South Jakarta, Kepulauan Seribu Regency), occupation (public, health worker, government staff), and history of previous COVID-19 infection (>3 months before the index PCR test). 95%CI: 95% Confidence Interval; VE: Vaccine Effectiveness.

Based on the unmatched analysis, the vaccine effectiveness (VE) of inactivated vaccines administered as 1-dose and 2-dose regimens during the Delta wave was 16.24% (95% CI 13.06–19.35) and 34.98% (95% CI 33.57–36.36), respectively. After conducting matched analysis and adjusting for other factors, the VE was found to be 1.92% (95% CI -2.47–6.20) for the 1-dose and 22.06% (95% CI 20.63–24.54) for the 2-dose inactivated vaccine (Fig 3). It appeared that the VE demonstrated mild protection against COVID-19 during the Delta wave for the 2-dose vaccines. The effectiveness of the inactivated vaccine during the Delta wave also varied based on the timing of the last vaccination, ranging from 6.66% to 32.67% for 1-dose and 30.50% to 56.08% for 2-dose (S1 Appendix, Fig B, Table C, and D). It was shown that the VE was acceptable when the assessment was conducted up to 28 days after the last dose, but it decreased after 28 days. On the other hand, the inactivated vaccine showed slightly higher protection against fatal COVID-19 throughout the Delta surge, as indicated by the results of unmatched analysis showing that the VE for 2 doses and 1 dose consecutively was 78.55% (95% CI 72.91–83.0) and 70.47% (95% CI 53.88–81.08), respectively (S1 Appendix Table E).

During the Omicron wave, the 2-dose inactivated vaccine showed negligible protection, with a VE of 4.52% (95% CI -0.90–9.61) in unmatched analysis and 0.80% (95% CI -6.01–7.50) in matched analysis after further adjustment for other factors. For boosters in the Omicron period, the inactivated vaccine also showed mild protection when the primary vaccine was an inactivated vaccine, with a VE of 24.99% (95% CI 22.97–26.95) in unmatched analysis and 26.66% (95% CI 24.27–28.97) after conducting a matched analysis and adjusting for other factors.

### Protection by mRNA-based platform vaccine

**Fig 4:**
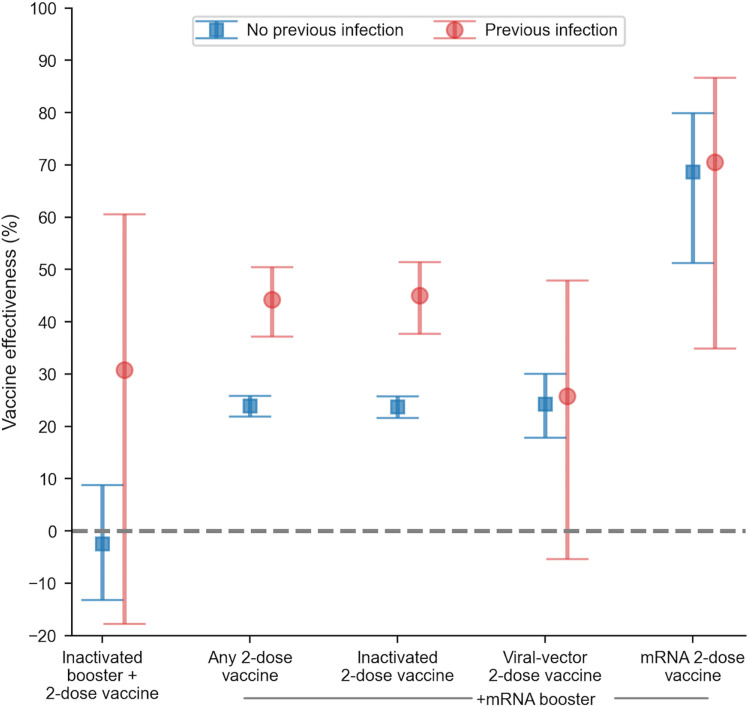
The VE against SARS-CoV-2 infection of booster regimen and 2-dose vaccines during Omicron period by history of previous COVID-19.

Data on the VE of mRNA vaccines were only available for the Omicron wave due to insufficient vaccine data during the Delta wave. The VE of the 2-dose mRNA vaccine against COVID-19 was 31.32% (95% CI 25.32–36.87) based on unmatched analysis and 24.81% (95% CI 16.81–32.09) based on matched analysis after further adjustment for other factors. The VE of mRNA vaccines against COVID-19 during the Omicron wave also varied over time, with the highest VE found in vaccines administered less than 14 days prior, at 53.28% (95% CI 32.36–67.73), and then tended to decrease over time (S1 Appendix, Fig C and Table D). From unmatched analysis, the mRNA vaccine as a booster also showed mild protection against COVID-19 with a VE of 25.10% (95% CI 23.13–27.02) regardless of the primary vaccine and good protection with a VE of 68.82% (95% CI 54.11–78.82) when the primary vaccine was an mRNA vaccine. In matched analysis with adjustment for other risk factors, the VE of the mRNA vaccine booster was found to be 23.81% (95% CI 21.34–28.82). Variations in VE were also presented based on the timing of the booster vaccine. The VE of the mRNA vaccine booster less than 7 days was 69.38% (95% CI 44.95–82.97), and for 7–14 days was 69.50% (95% CI 46.37–82.66) (S1 Appendix Fig B and Table F). For the mRNA vaccine booster with the primary vaccine also being mRNA, the VE was 70.54% (95% CI 34.95–86.65) in patients with a history of COVID-19, while the VE was 68.70% (95% CI 51.23–79.91) in patients without a history of COVID-19 (Fig 4).

## Discussion

### Inactivated vaccine

During the Delta wave, inactivated vaccines were the most widely used COVID-19 vaccines in LMICs, including Indonesia, while the availability of mRNA vaccines was still very limited [28]. The results of this study indicate that the VE of a 2-dose inactivated vaccine against infection in the Delta wave, conferred mild protection against COVID-19 (VE 35%). Our finding is consistent with a study conducted in Argentina (n= 237,330) using both TND and retrospective cohort design [29,30].

Furthermore, our study showed that 2-dose of inactivated vaccine did not protect against SARS-CoV-2 infection during Omicron wave VE 4.52% (95% CI -0.90 to 9.61). This result is similar to a prospective study in Hong Kong (n=7,750) that depicts VE of 2-dose CoronaVac against infection was 5.4% (95% CI –25.6–28.8) in the adult population [31].

Even though the vaccine design was based on the Spike Protein Receptor Binding Domain (S-RBD) of wild-type SARS-CoV-2 [32], the inactivated vaccine still had protection against SARS-CoV-2 infection during the delta period. S protein of Delta strain shows conformational changes compared to the wild-type virus [33] that leads to immune evasion [34]. However, CoronaVac vaccine induces S protein antibodies and prevents infection [35] and still has neutralization capacity against SARS-CoV-2 Delta variant infection [36]. Antibodies against protein S are also associated with the presence of IgG in saliva [37]. which is the first line of defense against infection, further demonstrating the role of mucosal immunity in viral infections. However, this differs from Omicron variants, which have approximately 30 mutations (from the virus), which lead to evading the immune system in Omicron BA.1 [38].

Even though the protection of the inactivated vaccine conferred mild protection in the Delta wave, the VE of 1-dose and the complete primary dose of the inactivated vaccine against fatal COVID-19 showed a value of 70.47% (95% CI 53.88–81.08) and 78.55% (95% CI 72.91–83.00), respectively. This result was in line with a study on the VE of CoronaVac against fatal cases, which showed a value of 87.4% [28] and a study in Hong Kong VE CoronaVac against fatal cases at 74.0% [39]. Relative VE (rVE) calculations showed that 2-dose provides 27.36% increased protection compared to 1-dose. With a relatively small increase in emergency public health conditions, instead of finishing the primary series, it could be possible to increase the coverage of the 1-dose vaccination to protect a more vulnerable group [40–42].

### mRNA vaccines

Our study demonstrated that the mRNA primary vaccine showed protection against Omicron variant infection with a VE of 24.81% (95%CI 16.81–32.09). Peak protection occurred <14 days after injection of the last dose with a VE of 53.28% (95%CI 32.36–67.73) and decreased continuously over time. Our study presents VE <14 days, 14–28 days, and 29–90 days of 53%, 32%, and <10%, respectively (S1 Appendix, Table D). A pattern observed in the VE of 2-dose mRNA against infection is similar in a systematic review and meta-analysis that showed VE of 2-dose mRNA against Omicron infection wane faster compared to Delta. The systematic review and meta-analysis showed the effectiveness of 3-dose of mRNA-1273 and BNT162b2 against infection 1 month, 3 months, 6 months, and 9 months after the last dose was 44–45%, 30–35%, 15–21%, and 7–15%, respectively [43].

Indonesian government policy for vaccination and booster programs applied half of the dose for booster vaccines for COVID-19 [11]. Even though this policy was supported by the results of a randomized control trial in Indonesia that half dose was comparable to a full dose in the context of immunogenicity, no reported study has proven the effectiveness of half-dose booster vaccines against infection. In our study, the result of VE of 3-dose mRNA booster in Omicron was 69% (S1 Appendix, Table D). In contrast, a study from Hong Kong revealed that VE against infection among naive population was 48% [44]. Our study demonstrates a higher and more sensible result due to the range of time of 7–90 days when compared to Hong Kong of 7–200 days. i.e., a narrower window for VE [44]. In our study, the result of VE of inactivated vaccine as primary with half-dose of mRNA vaccine as booster against infection was 26.7% (Fig 3). In a corresponding test-negative case-control study in the adult population of Colombia, the VE of non-mRNA primary vaccine and mRNA booster vaccine against infection was 31%, hence being comparable [45]. Therefore, a vaccination strategy using half-dose may benefit from constrained supply countries, which can extend booster vaccine coverage.

On the other hand, we can observe that the VE of 2-dose from our study and 3-dose from another study of inactivated vaccines did not confer protection against Omicron infection [46]. However, a combination of 2-dose of inactivated vaccine followed by a half-dose of an mRNA vaccine conferred protection against Omicron infection comparable to that of 2-dose of an mRNA vaccine. Therefore, for populations that have received inactivated vaccines as the primary series, the recommended booster option is a vaccine from a different platform, such as an mRNA vaccine.

The booster dose of the mRNA vaccine during the Omicron predominance period provided protection that appeared <7 days after injection and experienced a decreasing trend. Interestingly, the highest VE booster appeared in the population that received the homologous mRNA booster regimen [47]. Without considering the primary vaccine, the mRNA booster vaccine showed a VE against COVID-19 infection of 24% (21–29). This indicated that even though patients have received an inactivated primary vaccine, patients who then receive an mRNA booster vaccine may have mild protection against COVID-19, better than if patients receive an inactivated vaccine as their booster [46]. Additionally, people with a history of previous COVID-19 infection who received the mRNA vaccine from the primary series to the booster exhibited a high VE of 70.54% (95% CI 34.95–86.65), relatively similar to the VE observed in patients without a history of COVID-19, which was 68.70% (95% CI 51.23–79.91). A similar thing was also shown by a study in the USA [48].

## Strength and limitation

Our study is the first real-world VE study in Southeast Asian study with a sizable sample size that allows us to observe vaccine performance during the Delta and Omicron wave, which was the most catastrophic public health emergency in Indonesia. Even though unable to evaluate symptomatic or severity of the COVID-19 cases, which became a public health concern during the crisis, our study was able to evaluate vaccine performance regarding the protection against fatal COVID-19 (mortality within 30 days after RT-PCR SARS-CoV-2 confirmation). Our findings align with other studies, such as a global meta-analysis conducted in a real-world setting that has shown that approved vaccines are highly protective against SARS-CoV-2 [3]. Nevertheless, due to limited data availability, our study could not control for confounding factors like comorbidities and level of care received by infected subjects, significantly impacting COVID-19 transmission and case severity. Furthermore, data for this study only comes from Jakarta, one of the provinces in Indonesia with the highest and quickest rates of vaccination coverage. Therefore, our study findings may not be generalizable to the Indonesian population, for which vaccination uptake may vary [49].

Future research should focus on the long-term effectiveness of COVID-19 vaccines, particularly in maintaining immunity over extended periods and across diverse populations. Investigating the VE against emerging variants remains critical, as these variants may evade immunity, necessitating studies on how boosters can restore and enhance protection. Additionally, more research is needed to assess the durability of booster doses and the optimal timing for future boosters to ensure sustained immune responses.

## Conclusion

A complete primary dose of inactivated vaccine conferred mild protection against COVID-19, yet essential protection against fatal cases during the Delta wave. However, this vaccine conferred little to no protection during the Omicron wave. In contrast, the mRNA vaccine, either as primary vaccination, homologous, or heterologous booster regimen, conferred acceptable protection against Omicron infection. This study provided recommendations for real-world vaccination strategies in LMICs with typical source constraints for supplying vaccines to its population.

## Supporting information

S1 File Appendix. (DOCX)

## References

[1] Poland G, Levine M, Clemens J. Developing the next generation of vaccinologists. Vaccine. 2010;28(52):8227–8. 21129602 10.1016/j.vaccine.2010.11.001

[2] Watson OJ, Barnsley G, Toor J, Hogan AB, Winskill P, Ghani AC. Global impact of the first year of COVID-19 vaccination: a mathematical modelling study. Lancet Infect Dis. 2022;22(9):1293–302. doi: 10.1016/S1473-3099(22)00320-6 35753318 PMC9225255

[3] Zheng C, Shao W, Chen X, Zhang B, Wang G, Zhang W. Real-world effectiveness of COVID-19 vaccines: a literature review and meta-analysis. Int J Infect Dis. 2022;114:252–60. doi: 10.1016/j.ijid.2021.11.009 34800687 PMC8595975

[4] Zeng B, Gao L, Zhou Q, Yu K, Sun F. Effectiveness of COVID-19 vaccines against SARS-CoV-2 variants of concern: a systematic review and meta-analysis. BMC Med. 2022;20(1):200. doi: 10.1186/s12916-022-02397-y 35606843 PMC9126103

[5] Xu K, Wang Z, Qin M, Gao Y, Luo N, Xie W, et al. A systematic review and meta-analysis of the effectiveness and safety of COVID-19 vaccination in older adults. Front Immunol. 2023;14:1113156. doi: 10.3389/fimmu.2023.1113156 36936964 PMC10020204

[6] Ssentongo P, Ssentongo AE, Voleti N, Groff D, Sun A, Ba DM, et al. SARS-CoV-2 vaccine effectiveness against infection, symptomatic and severe COVID-19: a systematic review and meta-analysis. BMC Infect Dis. 2022;22(1):439. doi: 10.1186/s12879-022-07418-y 35525973 PMC9077344

[7] World Bank. Low & middle income [Internet]. World Bank; 2022 [cited 2023 Aug 23]. Available from: https://data.worldbank.org/country/XO

[8] Duan Y, Shi J, Wang Z, Zhou S, Jin Y, Zheng Z. Disparities in COVID-19 vaccination among low-, middle-, and high-income countries: the mediating role of vaccination policy. Vaccines (Basel). 2021;9(8):905. 34452030 10.3390/vaccines9080905 PMC8402650

[9] Duroseau B, Kipshidze N, Limaye RJ. The impact of delayed access to COVID-19 vaccines in low- and lower-middle-income countries. Front Public Health. 2023;10:1087138. doi: 10.3389/fpubh.2022.1087138 36711400 PMC9878283

[10] President of the Republic of Indonesia. Presidential Regulation No. 99 of 2020 regarding procurement of vaccines and implementation of vaccinations in the context of overcoming the Coronavirus Disease 2019 (COVID-19) pandemic Vol LN.2020/No.227. Jakarta; 2020.

[11] Ministry of Health Republic of Indonesia. Notification Letter No. HK.02.02/II/252/2022 concerning advanced dose (Booster) COVID-19 vaccination. Jakarta; 2022.

[12] King C, Beard J, Crampin A, Costello A, Mwansambo C, Cunliffe N. Methodological challenges in measuring vaccine effectiveness using population cohorts in low resource settings. Vaccine. 2015;33(38):4748–55. 26235370 10.1016/j.vaccine.2015.07.062 PMC4570930

[13] World Health Organization. Evaluation of COVID-19 vaccine effectiveness: interim guidance, 17 March 2021 (No. WHO/2019-nCoV/vaccine_effectiveness/measurement/2021.1). 2021.

[14] Jackson ML, Nelson JC. The test-negative design for estimating influenza vaccine effectiveness. Vaccine. 2013;31(17):2165–8. doi: 10.1016/j.vaccine.2013.02.053 23499601

[15] Fukushima W, Hirota Y. Basic principles of test-negative design in evaluating influenza vaccine effectiveness. Vaccine. 2017;35(36):4796–800. 28818471 10.1016/j.vaccine.2017.07.003

[16] Chemaitelly H, Tang P, Hasan M, AlMukdad S, Yassine H, Benslimane F, et al. Waning of BNT162b2 vaccine protection against SARS-CoV-2 infection in Qatar. New Eng J Med. 2021. doi: 10.1056/NEJMoa2114114 PMC8522799 34614327

[17] Chemaitelly H, Yassine HM, Benslimane FM, Al Khatib HA, Tang P, Hasan MR, et al. mRNA-1273 COVID-19 vaccine effectiveness against the B.1.1.7 and B.1.351 variants and severe COVID-19 disease in Qatar. Nat Med. 2021 Sep;27(9):1614–21. 34244681 10.1038/s41591-021-01446-y

[18] Costa-Santos C, Neves AL, Correia R, Santos P, Monteiro-Soares M, Freitas A, et al. COVID-19 surveillance data quality issues: a national consecutive case series. BMJ Open. 2021;11(12):e047623. doi: 10.1136/bmjopen-2020-047623 34872992 PMC8649880

[19] Ioannidis JPA, Zonta F, Levitt M. Flaws and uncertainties in pandemic global excess death calculations. Eur J Clin Invest. 2023;53(8):e14008. doi: 10.1111/eci.14008 37067255 PMC10404446

[20] Msemburi W, Karlinsky A, Knutson V, Aleshin-Guendel S, Chatterji S, Wakefield J. The WHO estimates of excess mortality associated with the COVID-19 pandemic. Nature. 2023;613(7942):130–7. doi: 10.1038/s41586-022-04804-5 36517599 PMC9812776

[21] Brownson RC, Burke TA, Colditz GA, Samet JM. Reimagining public health in the aftermath of a pandemic. Am J Public Health. 2020;110(11):1605–10. doi: 10.2105/AJPH.2020.305861 32816552 PMC7542265

[22] Sidky H, Young JC, Girvin AT, Lee E, Shao YR, Hotaling N, et al. Data quality considerations for evaluating COVID-19 treatments using real world data: learnings from the National COVID Cohort Collaborative (N3C). BMC Med Res Methodol. 2023;23(1):46. doi: 10.1186/s12874-023-01839-2 36800930 PMC9936475

[23] World Health Organization. Evaluation of COVID-19 vaccine effectiveness in a changing landscape of COVID-19 epidemiology and vaccination: interim guidance, 3 October 2022 (No. WHO/2019-nCoV/vaccine_effec- tiveness/VE_evaluations/2022.1). 2022.

[24] Austin P. Using the standardized difference to compare the prevalence of a binary variable between two groups in observational research. Commun Stat- Simul Comput. 2009;38(6):1228–34.

[25] Kumar R, Chhabra P. Cautions required during planning, analysis and reporting of multivariable logistic regression. Curr Med Res Pract. 2014;4(1):31–9.

[26] Zhang Y-Y, Zhou X-B, Wang Q-Z, Zhu X-Y. Quality of reporting of multivariable logistic regression models in Chinese clinical medical journals. Medicine (Baltimore). 2017;96(21):e6972. doi: 10.1097/MD.0000000000006972 28538397 PMC5457877

[27] Seabold S, Perktold J. Statsmodels: econometric and statistical modeling with python. 2010.

[28] Suryatma A, Anasi R, Hananto M, Hermawan A, Ramadhany R, Indalao IL, et al. Effectiveness of the inactivated COVID-19 Vaccine (CoronaVac) in adult population in Bali, Indonesia [Internet]. medRxiv; 2022 [cited 2024 Jan 29]. p. 2022.02.02.22270351. Available from: https://www.medrxiv.org/content/10.1101/2022.02.02.22270351v1

[29] Li X, Yang X, Ning Z. Efficacy and safety of COVID-19 inactivated vaccine: a meta-analysis. Front Med (Lausanne). 2022;9:1015184. doi: 10.3389/fmed.2022.1015184 36419789 PMC9676443

[30] Law M, Ho S, Tsang G, Ho C, Kwan C, Yan V. Efficacy and effectiveness of inactivated vaccines against symptomatic COVID-19, severe COVID-19, and COVID-19 clinical outcomes in the general population: a systematic review and meta-analysis. Lancet Regional Health - Western Pacific. 2023;37:100788. 37360863 10.1016/j.lanwpc.2023.100788 PMC10199328

[31] Effectiveness of BNT162b2 and CoronaVac COVID-19 vaccination against asymptomatic and symptomatic infection of SARS-CoV-2 omicron BA.2 in Hong Kong: a prospective cohort study - The Lancet Infectious Diseases [Internet]. [cited 2024 Sep 15]. Available from: https://www.thelancet.com/journals/laninf/article/PIIS1473-3099(22)00732-0/fulltext 10.1016/S1473-3099(22)00732-0 PMC9744442 36521506

[32] Tai W, He L, Zhang X, Pu J, Voronin D, Jiang S, et al. Characterization of the receptor-binding domain (RBD) of 2019 novel coronavirus: implication for development of RBD protein as a viral attachment inhibitor and vaccine. Cell Mol Immunol. 2020;17(6):613–20. doi: 10.1038/s41423-020-0400-4 32203189 PMC7091888

[33] Mahmood TB, Hossan MI, Mahmud S, Shimu MSS, Alam MJ, Bhuyan MMR, et al. Missense mutations in spike protein of SARS-CoV-2 delta variant contribute to the alteration in viral structure and interaction with hACE2 receptor. Immun Inflamm Dis. 2022;10(9):e683. doi: 10.1002/iid3.683 36039645 PMC9382871

[34] McCallum M, Walls AC, Sprouse KR, Bowen JE, Rosen LE, Dang HV, et al. Molecular basis of immune evasion by the Delta and Kappa SARS-CoV-2 variants. Science. 2021;374(6575):1621–6. doi: 10.1126/science.abl8506 34751595 PMC12240541

[35] Azak E, Karadenizli A, Uzuner H, Karakaya N, Canturk NZ, Hulagu S. Comparison of an inactivated Covid19 vaccine-induced antibody response with concurrent natural Covid19 infection. Int J Infect Dis. 2021;113:58–64. doi: 10.1016/j.ijid.2021.09.060 34597764 PMC8479817

[36] Yadav P, Sapkal G, Ella R, Sahay R, Nyayanit D, Patil D, et al. Neutralization against B.1.351 and B.1.617.2 with sera of COVID-19 recovered cases and vaccinees of BBV15 [Internet]. 2021 [cited 2024 Jan 29]. Available from: https://europepmc.org/article/PPR/PPR353180 10.1093/jtm/taab104 PMC8344909 34230972

[37] Li D, Calderone R, Nsouli TM, Reznikov E, Bellanti JA. Salivary and serum IgA and IgG responses to SARS-CoV-2-spike protein following SARS-CoV-2 infection and after immunization with COVID-19 vaccines. Allergy Asthma Proc. 2022;43(5):419–30. doi: 10.2500/aap.2022.43.220045 36065108 PMC9465644

[38] Tian D, Sun Y, Zhou J, Ye Q. The global epidemic of SARS-CoV-2 variants and their mutational immune escape. J Med Virol. 2022;94(3):847–57. doi: 10.1002/jmv.27376 34609003 PMC8661756

[39] Wei Y, Jia K, Zhao S, Hung C, Mok C, Poon P. Estimation of vaccine effectiveness of CoronaVac and BNT162b2 against severe outcomes over time among patients with SARS-CoV-2 Omicron. JAMA Network Open. 2023;6(2):e2254777. 10.1001/jamanetworkopen.2022.54777 PMC9898822 36735253

[40] Aguas R, Bharath A, White LJ, Gao B, Pollard AJ, Voysey M, et al. Potential global impacts of alternative dosing regimen and rollout options for the ChAdOx1 nCoV-19 vaccine. Nat Commun. 2021;12(1):6370. doi: 10.1038/s41467-021-26449-8 34737262 PMC8569205

[41] Lu L, Zhang H, Zhan M, Jiang J, Yin H, Dauphars D. Preventing mortality in COVID-19 patients: which cytokine to target in a raging storm? Front Cell Develop Biol. 2020;8:677. 10.3389/fcell.2020.00677 PMC7379422 32766256

[42] Sun Y, Kang H, Zhao Y, Cui K, Wu X, Huang S. Immune response after vaccination using inactivated vaccine for coronavirus disease 2019. Chin Med J (English). 2023;136(12):1497–9. 10.1097/CM9.0000000000002707 PMC10278726 37154087

[43] Evaluation of waning of SARS-CoV-2 vaccine–induced immunity: a systematic review and meta-analysis | Infectious Diseases | JAMA Network Open | JAMA Network [Internet]. [cited 2024 Sep 15]. Available from: https://jamanetwork.com/journals/jamanetworkopen/fullarticle/2804451 10.1001/jamanetworkopen.2023.10650 PMC10157431 37133863

[44] Effectiveness of BNT162b2 and CoronaVac COVID-19 vaccination against asymptomatic and symptomatic infection of SARS-CoV-2 omicron BA.2 in Hong Kong: a prospective cohort study - The Lancet Infectious Diseases [Internet]. [cited 2024 Sep 15]. Available from: https://www.thelancet.com/journals/laninf/article/PIIS1473-3099(22)00732-0/fulltext 10.1016/S1473-3099(22)00732-0 PMC9744442 36521506

[45] Heterologous and BNT162b2 boosters are more effective than non-mRNA homologous boosters for Omicron - Journal of Infection [Internet]. [cited 2024 Sep 15]. Available from: https://www.journalofinfection.com/article/S0163-4453(24)00099-9/fulltext 10.1016/j.jinf.2024.106165 38670269

[46] Omicron breakthrough infections after triple‐dose inactivated COVID‐19 vaccination: a comprehensive analysis of antibody and T‐cell responses - Xiang - 2024 - Immunology - Wiley Online Library [Internet]. [cited 2024 Sep 15]. Available from: https://onlinelibrary.wiley.com/doi/full/10.1111/imm.13764 10.1111/imm.13764 38462236

[47] Wu N, Joyal-Desmarais K, Ribeiro PAB, Vieira AM, Stojanovic J, Sanuade C, et al. Long-term effectiveness of COVID-19 vaccines against infections, hospitalisations, and mortality in adults: findings from a rapid living systematic evidence synthesis and meta-analysis up to December, 2022. Lancet Respir Med. 2023;11(5):439–52. doi: 10.1016/S2213-2600(23)00015-2 36780914 PMC9917454

[48] Lind ML, Robertson AJ, Silva J, Warner F, Coppi AC, Price N, et al. Effectiveness of primary and booster COVID-19 mRNA vaccination against Omicron variant SARS-CoV-2 infection in people with a prior SARS-CoV-2 infection [Internet]. medRxiv; 2022 [cited 2024 Jan 29]. p. 2022.04.19.22274056. Available from: https://www.medrxiv.org/content/10.1101/2022.04.19.22274056v3 10.1371/journal.pmed.1004136 PMC9714718 36454733

[49] Harapan BN, Harapan T, Theodora L, Anantama NA. From archipelago to pandemic battleground: unveiling Indonesia’s COVID-19 crisis. J Epidemiol Glob Health. 2023;13(4):591–603. doi: 10.1007/s44197-023-00148-7 37707715 PMC10686963

