# Supplementary material for: Vaccine effectiveness of inactivated and mRNA COVID-19 vaccine platform during Delta and Omicron wave in Jakarta, Indonesia: A test-negative case-control study
Source: PLoS One. 2025 Jun 9;20(6):e0320779. doi: 10.1371/journal.pone.0320779 (PMC12148159; doi:10.1371/journal.pone.0320779)
Supplement: S1 File — (DOCX) [file pone.0320779.s001.docx]

# Appendix


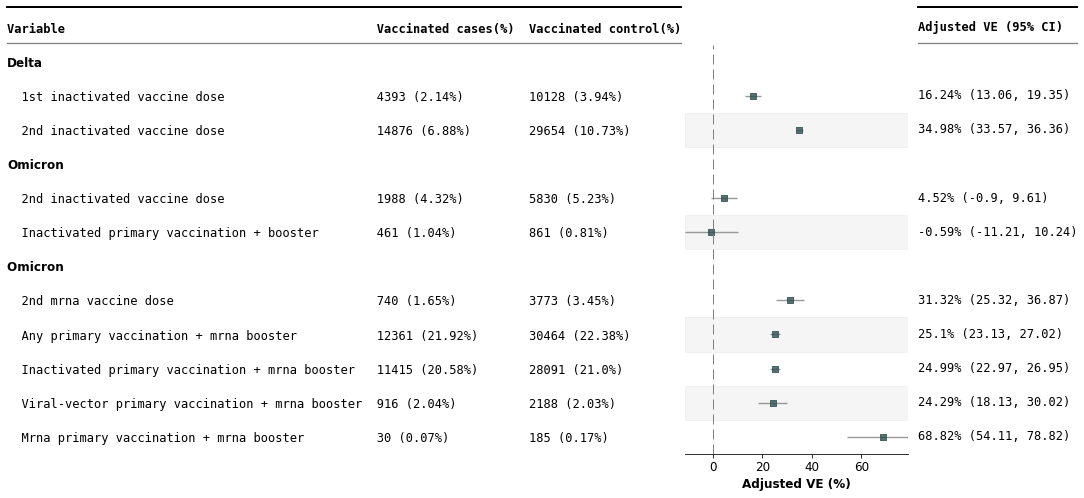


**Fig A. Forest plot for the vaccine effectiveness of Inactivated and mRNA vaccine against COVID-19 during Delta and Omicron Wave: Unmatched Analysis**


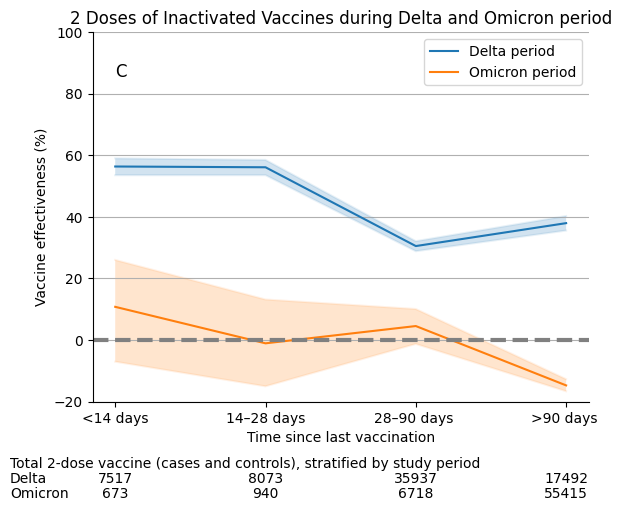


**Fig B. Vaccine effectiveness of 2-dose Inactivated vaccine against COVID-19 by time during Delta and Omicron Wave**


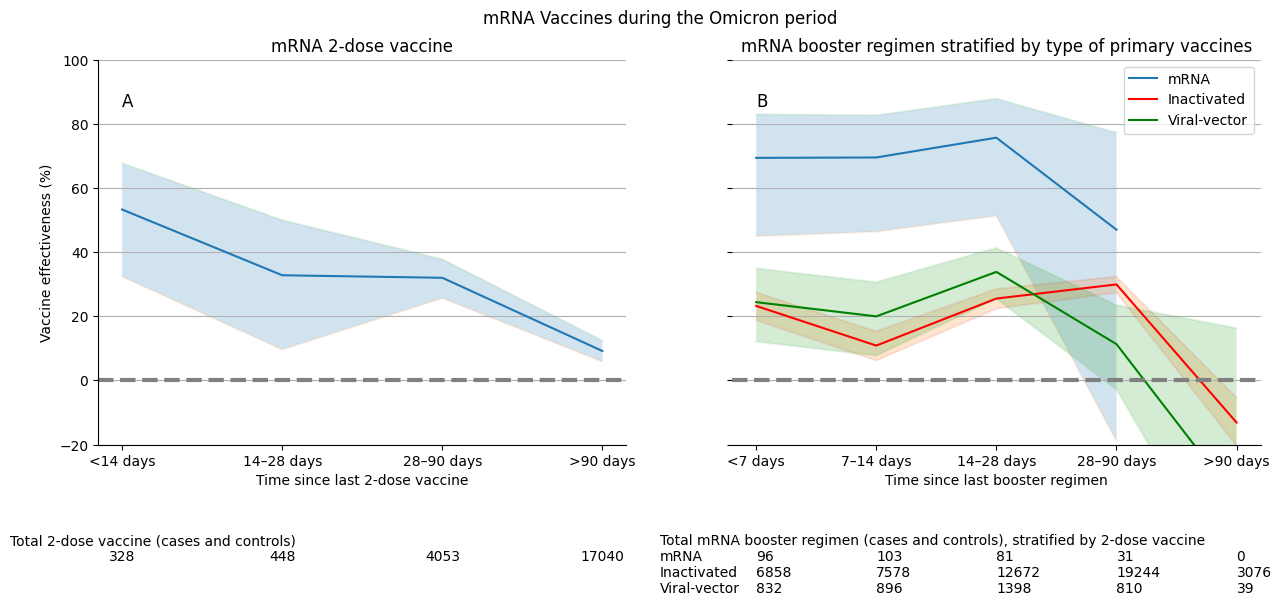


**Fig C. Vaccine effectiveness of 2-dose mRNA vaccine against COVID-19 (left) and mRNA booster regimen stratified by type of primary vaccine (right) by time during Omicron Wave**

**Table A. Vaccine effectiveness of Inactivated and mRNA vaccine against COVID-19 during Delta and Omicron Wave: Unmatched Analysis**

| **Vaccine type evaluated** | **Delta wave (1 June – 31 August 2021)** | | | | | | **Omicron wave (1 January – 2 April 2022)** | | | | | |
| --- | --- | --- | --- | --- | --- | --- | --- | --- | --- | --- | --- | --- |
|  | **Total Cases** | | **Total Control** | | **OR*  (95%CI)** | **VE*  (95%CI)** | **Total Cases** | | **Total Control** | | **OR*  (95%CI)** | **VE*  (95%CI)** |
|  | **Vax +** | **Vax -** | **Vax +** | **Vax -** |  |  | **Vax +** | **Vax -** | **Vax +** | **Vax -** |  |  |
| **1-dose of vaccines^a^** | | | | | | | | | | | | |
| Inactivated vaccines | 4,393 | 201,351 | 10,128 | 246,794 | 0.84  (0.81–0.87) | 16.24% (13.06–19.35) |  | | | | | |
| **2-dose of vaccines^b^** | | | | | | | | | | | | |
| Inactivated vaccines | 14,876 | 201,351 | 29,654 | 246,794 | 0.65  (0.64–0.66) | 34.98% (33.57–36.36) | 1,988 | 44,038 | 5,830 | 105,684 | 0.95  (0.90–1.01) | 4.52%  (-0.90 to 9.61) |
| mRNA vaccines | 1 | 201,351 | 2 | 246,794 | NA | NA | 740 | 44,038 | 3,773 | 105,684 | 0.69  (0.63–0.75) | 31.32% (25.32–36.87) |
| **Booster Regimen^c^** | | | | | | | | | | | | |
| Inactivated Booster dose  (regardless the primary vaccines) | 0 | 201,35-1 | 0 | 246,794 | NA | NA | 461 | 44,038 | 861 | 105,684 | 1.01  (0.90–1.13) | -0.59%  (-11.21 to 10.24) |
| mRNA booster dose  (regardless the primary vaccines) | 3 | 201,351 | 80 | 246,794 | NA | NA | 12,361 | 44,038 | 30,464 | 105,684 | 0.75  (0.73–0.77) | 25.10% (23.13–27.02) |
| Inactivated primary vaccines | 3 | 201,351 | 80 | 246,794 | NA | NA | 11,415 | 44,038 | 28,091 | 105,684 | 0.75  (0.73–0.77) | 24.99% (22.97–26.95) |
| ChAdOX1 nCoV-19 primary vaccines | 0 | 201,351 | 0 | 246,794 | NA | NA | 916 | 44,038 | 2,188 | 105,684 | 0.76  (0.77–0.82) | 24.29% (18.13–30.02) |
| mRNA primary vaccines | 0 | 201,351 | 0 | 246,794 | NA | NA | 30 | 44,038 | 185 | 105,684 | 0.31  (0.21–0.46) | 68.82% (54.11–78.82) |

*Adjusted to gender, age (<45 years and ≥45 years), domicile (Central Jakarta, West Jakarta, North Jakarta, East Jakarta, South Jakarta, Kepulauan Seribu), occupation (public, health worker, government staff), week of PCR test, and history of previous COVID-19 infection (>3 months before the index PCR test)

^a^ Range time since last dose 1 is 14–90 days before the index PCR test

^b^ Range time since last dose 2 is 14–90 days before the index PCR test

^c^Time since last booster is 7–90 days

95%CI: 95% Confidence Interval; OR: Odds ratio; Vax+ : Vaccinated; Vax-: Unvaccinated; VE: Vaccine effectiveness

**Table B. Vaccine effectiveness of Inactivated and mRNA vaccine against COVID-19 during Delta and Omicron Wave: Matched Analysis**

| **Vaccine type evaluated** | **Delta wave (1 June – 31 August 2021)** | | | | | | **Omicron wave (1 January – 2 April 2022)** | | | | | | |
| --- | --- | --- | --- | --- | --- | --- | --- | --- | --- | --- | --- | --- | --- |
|  | **Total Cases** | | **Total Control** | | **With matching factors^d^** | **Further adjusted with other factors*** | **Total Cases** | | | **Total Control** | | **With matching factors^e^** | **Further adjusted with other factors*** |
|  | **Vax +** | **Vax -** | **Vax +** | **Vax -** |  |  | **Vax +** | **Vax -** | | **Vax +** | **Vax -** |  |  |
| **1**-**dose^a^** | | | | | | | | | | | | | |
| Inactivated | 13,254 | 177,908 | 17,604 | 173,558 | 5.46%  (1.19–9.52) | 1.92%  (-2.47 to 6.20) |  | | | | | | |
| **2-dose^b^** | | | | | | | | | | | | | |
| Inactivated | 13,254 | 177,908 | 17,604 | 173,558 | 30.09% | 22.06%  (20.63–24.54) | 1,229 | | 26,316 | 2,639 | 52,541 | 7.23% (0.50–13.50) | 0.80% (-6.01 to 7.50) |
| mRNA |  | | | | | | 531 | | 28,082 | 1,509 | 55,717 | 30.52% (23.20–37.19) | 24.81% (16.81–32.09) |
| **Booster Regimen**^c^ | | | | | | | | | | | | | |
| Inactivated Booster dose  (regardless the primary vaccines) |  | | | | | | 395 | | 35,005 | 692 | 70,108 | -12.73% (-22.97 to -1.09) | -12.51% (-22.82 to -0.80) |
| mRNA booster dose  (regardless the primary vaccines) |  | | | | | | 8,384 | | 29,751 | 19,757 | 56,513 | 22.45% (19.99–24.87) | 23.81% (21.34–28.82) |
| Inactivated primary vaccines |  | | | | | | 8,122 | | 31,403 | 19,616 | 59,434 | 25.21% (22.82–27.53) | 26.66% (24.27–28.97) |
| ChAdOX1 nCoV-19 primary vaccines |  | | | | | | 679 | | 27,764 | 1,347 | 55,539 | -0.74% (-9.79 to 8.42) | 1.43% (-7.78 to 10.42) |
| mRNA primary vaccines |  | | | | | | 28 | | 29,634 | 127 | 59,197 | NA | NA |

^a^ Range time since last dose 1 is 14–90 days before the index PCR test

^b^ Range time since last dose 2 is 14–90 days before the index PCR test

^c^Time since last booster is 7–90 days

^d^Case and controls were matched exactly in a 1:1 ratio according to gender, 10-year age group, calendar week of PCR test,

^e^Case and controls were matched exactly in a 1:2 ratio according to gender, 10-year age group, calendar week of PCR test,

*Adjusted to domicile (Central Jakarta, West Jakarta, North Jakarta, East Jakarta, South Jakarta, Kepulauan Seribu), occupation (public, health worker, government staff), week of PCR test, and history of previous COVID-19 infection (>3 months before the index PCR test)

95%CI: 95% Confidence Interval; OR: Odds ratio; Vax +: Vaccinated; Vax-: Unvaccinated; VE: Vaccine effectiveness

**Table C. Vaccine effectiveness of 1 dose Inactivated vaccine against COVID-19 by time during Delta Wave**

| **Vaccine type evaluated** | **Delta wave (1 June – 31 August 2021)** | | | | | |
| --- | --- | --- | --- | --- | --- | --- |
|  | **Total Cases** | | **Total Control** | | **OR*  (95%CI)** | **VE*  (95%CI)** |
|  | **Vax +** | **Vax -** | **Vax +** | **Vax -** |  |  |
| <14 days after last dose | 2,202 | 201,351 | 5,023 | 246,794 | 0.67 (0.64–0.71) | 32.67% (29.04–36.11) |
| 14–28 days after last dose | 2,473 | 201,351 | 3,462 | 246,794 | 0.78 (0.74–0.82) | 21.83% (17.88–25.62) |
| 29–56 days after last dose | 1,357 | 201,351 | 3,311 | 246,794 | 0.93 (0.87–1.00) | 6.66% (0.30–12.63) |
| 57–90 days after last dose | 537 | 201,351 | 815 | 246,794 | 0.86 (0.77–0.96) | 14.08% (3.92–23.20) |
| >90 days after last dose | 758 | 201,351 | 1,552 | 246,794 | 0.78 (0.71–0.85) | 22.28% (14.96–28.97) |

*Adjusted to gender, age (<45 years and ​​≥45 years), domicile (Central Jakarta, West Jakarta, North Jakarta, East Jakarta, South Jakarta, Kepulauan Seribu), occupation (public, health worker, government staff), week of PCR test, and history of previous COVID-19 infection (>3 months before the index PCR test)

95%CI: 95% Confidence Interval; OR: Odds ratio; Vax+: Vaccinated; Vax-: Unvaccinated; VE: Vaccine effectiveness

**Table D. Vaccine effectiveness of 2-dose Inactivated and mRNA vaccine against COVID-19 by time during Delta and Omicron Wave**

| **Vaccine type evaluated** | **Delta wave (1 June – 31 August 2021)** | | | | | | **Omicron wave (1 January – 2 April 2022)** | | | | | |
| --- | --- | --- | --- | --- | --- | --- | --- | --- | --- | --- | --- | --- |
|  | **Total Cases** | | **Total Control** | | **OR*  (95%CI)** | **VE*  (95%CI)** | **Total Cases** | | **Total Control** | | **OR*  (95%CI)** | **VE*  (95%CI)** |
|  | **Vax +** | **Vax -** | **Vax +** | **Vax -** |  |  | **Vax +** | **Vax -** | **Vax +** | **Vax -** |  |  |
| **<14 days after last dose** | | | | | | | | | | | | |
| inactivated vaccines | 1,271 | 201,351 | 6,246 | 246,794 | 0.46  (0.41–0.44) | 56.33%  (53.51–58.98) | 159 | 44,038 | 514 | 105,684 | 0.89  (0.74–1.08) | 10.77%  (-7.04 to 25.99) |
| mRNA vaccines | NA | NA | NA | NA | NA | NA | 75 | 44,038 | 253 | 105,684 | 0.47  (0.32–0.68) | 53.28%  (32.36–67.73) |
| **14–28 days after last dose** | | | | | | | | | | | | |
| inactivated vaccines | 1,585 | 201,351 | 6,488 | 246,794 | 0.44  (0.42–0.46) | 56.08%  (53.51 –58.48) | 251 | 44,038 | 689 | 105,684 | 1.01  (0.87–1.18) | -1.08%  (-15.04 to 13.15) |
| mRNA vaccines | NA | NA | NA | NA | NA | NA | 91 | 44,038 | 357 | 105,684 | 0.67  (0.50–0.90) | 32.79%   (9.61–49.99) |
| **29–90 days after last dose** | | | | | | | | | | | | |
| inactivated vaccines | 13,093 | 201,351 | 22,844 | 246,794 | 0.69  (0.68–0.71) | 30.50%  (28.82–32.09) | 1,704 | 44,038 | 5,014 | 105,684 | 0.95  (0.90–1.01) | 4.53%  (-1.29 to 10.06) |
| mRNA vaccines | NA | NA | NA | NA | NA | NA | 644 | 44,038 | 3,409 | 105,684 | 0.68  (0.62–0.74) | 32.00%  (25.62–37.81) |
| **>90 days after last dose** | | | | | | | | | | | | |
| inactivated vaccines | 4,079 | 201,351 | 13,413 | 246,794 | 0.62 (0.60–0.64) | 37.94%  (35.53–40.25) | 15,739 | 44,038 | 39,676 | 105,684 | 1.17 (1.15–1.20) | -14.74%  (-16.64 to -12.72) |
| mRNA vaccines | NA | NA | NA | NA | NA | NA | 4,971 | 44,038 | 12,069 | 105,684 | 0.91 (0.88–0.94) | 9.17%  (5.82–12.45) |

*Adjusted to gender, age (<45 years and ≥45 years), domicile (Central Jakarta, West Jakarta, North Jakarta, East Jakarta, South Jakarta, Kepulauan Seribu), occupation (public, health worker, government staff), week of PCR test, and history of previous COVID-19 infection (>3 months before the index PCR test)

95%CI: 95% Confidence Interval; OR: Odds ratio; Vax +: Vaccinated; Vax-: Unvaccinated; VE: Vaccine effectiveness

**Table E. Vaccine effectiveness of Inactivated vaccine against Fatal COVID-19 during Delta Wave**

| **Vaccine type evaluated** | **Delta wave (1 June – 31 August 2021)** | | | | | |
| --- | --- | --- | --- | --- | --- | --- |
|  | **Total Cases** | | **Total Control** | | **OR*  (95%CI)** | **VE*  (95%CI)** |
|  | **Vax +** | **Vax -** | **Vax +** | **Vax -** |  |  |
| **1-dose of Inactivated vaccines^a^** | 20 | 2,228 | 10,128 | 246,794 | 0.30 (0.19–0.46) | 70.47% (53.88–81.08) |
| **2-dose of Inactivated vaccines^b^** | 74 | 2,228 | 29,654 | 246,794 | 0.21 (0.17–0.27) | 78.55% (72.91–83.00) |

*Adjusted to gender, age (<45 years and >=45 years), domicile (Central Jakarta, West Jakarta, North Jakarta, East Jakarta, South Jakarta, Kepulauan Seribu), occupation (public, health worker, government staff), week of PCR test, and history of previous COVID-19 infection (>3 months before the index PCR test)

^a^ Range time since last dose 1 is 14–90 days before the index PCR test

^b^ Range time since last dose 2 is 14–90 days before the index PCR test

95%CI: 95% Confidence Interval; OR: Odds ratio; Vax+ : Vaccinated; Vax-: Unvaccinated; VE: Vaccine effectiveness

**Table F. Vaccine effectiveness of mRNA vaccine booster against COVID-19 by time during Omicron Wave**

| **Primary vaccine type evaluated** | **Omicron wave (1 January – 2 April 2022)** | | | | | |
| --- | --- | --- | --- | --- | --- | --- |
|  | **Total Cases** | | **Total Control** | | **OR*  (95%CI)** | **VE*  (95%CI)** |
|  | **Vax +** | **Vax -** | **Vax +** | **Vax -** |  |  |
| **<7 days after last booster** | 2,102 | 44,038 | 5,684 | 105,684 | 0.76 (0.72–0.80) | 24.08% (19.99–27.96) |
| + inactivated | 1,846 | 44,038 | 5,012 | 105,684 | 0.77 (0.73–0.81) | 23.23% (18.78–27.46) |
| + ChAdOX1 nCoV-19 | 243 | 44,038 | 589 | 105,684 | 0.76 (0.65–0.88) | 24.41% (12.10–34.95) |
| + mRNA | 13 | 44,038 | 83 | 105,684 | 0.31 (0.17–0.55) | 69.38% (44.95–82.97) |
| **7–14 days after last booster** | 2,738 | 44,038 | 5,839 | 105,684 | 0.87 (0.83–0.91) | 12.76% (8.52–16.89) |
| + inactivated | 2,449 | 44,038 | 5,129 | 105,684 | 0.89 (0.85–0.94) | 10.87% (6.20–15.30) |
| + ChAdOX1 nCoV-19 | 275 | 44,038 | 621 | 105,684 | 0.80 (0.69–0.92) | 19.97% (7.69–30.65) |
| + mRNA | 14 | 44,038 | 89 | 105,684 | 0.30 (0.17–0.54) | 69.50% (46.37–82.66) |
| **15–28 days after last booster** | 4,056 | 44,038 | 10,095 | 105,684 | 0.73 (0.70–0.76) | 26.73% (23.81–29.53) |
| + inactivated | 3,673 | 44,038 | 8,999 | 105,684 | 0.74 (0.71–0.78) | 25.53% (22.35–28.54) |
| + ChAdOX1 nCoV-19 | 374 | 44,038 | 1,024 | 105,684 | 0.66 (0.59–0.75) | 33.84% (25.47–41.32) |
| + mRNA | 9 | 44,038 | 72 | 105,684 | NA | NA |
| **29–90 days after last booster** | 5,562 | 44,038 | 14,523 | 105,684 | 0.71 (0.68–0.73) | 29.11% (26.58–31.55) |
| + inactivated | 5,288 | 44,038 | 13,956 | 105,684 | 0.70 (0.68–0.73) | 29.93% (27.39–32.36) |
| + ChAdOX1 nCoV-19 | 267 | 44,038 | 543 | 105,684 | 0.89 (0.76–1.06) | 11.33% (-2.76 to 23.51) |
| + mRNA | 7 | 44,038 | 24 | 105,684 | NA | NA |
| **>90 days after last booster** | 1,102 | 44,038 | 2,013 | 105,684 | 1.16 (1.06–1.26) | -13.59% (-20.71 to -5.92) |
| + inactivated | 1,082 | 44,038 | 1,994 | 105,684 | 1.15 (1.06–1.25) | -13.11% (-20.31 to -5.35) |
| + ChAdOX1 nCoV-19 | 20 | 44,038 | 19 | 105,684 | NA | NA |
| + mRNA | 0 | 44,038 | 0 | 105,684 | NA | NA |

*Adjusted to gender, age (<45 years and ≥45 years), domicile (Central Jakarta, West Jakarta, North Jakarta, East Jakarta, South Jakarta, Kepulauan Seribu), occupation (public, health worker, government staff), week of PCR test, and history of previous COVID-19 infection (>3 months before the index PCR test)

95%CI: 95% Confidence Interval; OR: Odds ratio; Vax+ : Vaccinated; Vax-: Unvaccinated; VE: Vaccine effectiveness

**Table G. Vaccine effectiveness of mRNA vaccine against COVID-19 during Omicron Wave by History of previous COVID-19**

| **Vaccine type evaluated** | **Omicron wave (1 January – 2 April 2022)** | | | | |
| --- | --- | --- | --- | --- | --- |
|  | **Total Cases** | | **Total Control** | | **VE*  (95%CI)** |
|  | **Vax +** | **Vax -** | **Vax +** | **Vax -** |  |
| **2-dose of Inactivated vaccines^a^** | | | | | |
| History of COVID-19 infection^b^ | 376 | 2,727 | 1,143 | 8,405 | -8.82% (-20.15 to 4.02) |
| No History of COVID-19 infection | 1,612 | 41,311 | 4,687 | 97,279 | 7.63% (1.88 –13.06) |
| **Inactivated Booster dose^c^** | | | | | |
| History of COVID-19 infection^b^ | 16 | 2,727 | 54 | 8,405 | 30.75% (-17.72 to 60.54) |
| No History of COVID-19 infection | 445 | 41,311 | 807 | 97,279 | -2.43% (-13.15 to 8.79) |
| **2-dose of mRNA vaccines^a^** | | | | | |
| History of COVID-19 infection^b^ | 167 | 2,727 | 909 | 8,405 | 21.87% (6.29–34.88) |
| No History of COVID-19 infection | 573 | 41,311 | 2,864 | 97,279 | 33.68% (27.09–39.71) |
| **mRNA Booster dose^c^** | | | | | |
| History of COVID-19 infection^b^ | 467 | 2,727 | 2,020 | 8,405 | 44.19% (37.19–50.44) |
| No History of COVID-19 infection | 11,894 | 41,311 | 28,444 | 97,279 | 23.90% (21.89–25.84) |
| **+ inactivated primary vaccines** | | | | | |
| History of COVID-19 infection^b^ | 418 | 2,727 | 1,841 | 8,405 | 45.01% (37.69–51.47) |
| No History of COVID-19 infection | 10,997 | 41,311 | 26,250 | 97,279 | 23.75% (21.65–25.77) |
| **+ ChAdOX1 nCoV-19 primary vaccines** | | | | | |
| History of COVID-19 infection^b^ | 42 | 2,727 | 128 | 8,405 | 25.80% (-5.35 to 47.90) |
| No History of COVID-19 infection | 874 | 41,311 | 2,060 | 97,279 | 24.24% (17.88–30.09) |
| **+ mRNA primary vaccines** | | | | | |
| History of COVID-19 infection^b^ | 7 | 2,727 | 51 | 8,405 | 70.54% (34.95–86.65) |
| No History of COVID-19 infection | 23 | 41,311 | 134 | 97,279 | 68.70% (51.23–79.91) |

*Adjusted to gender, age (<45 years and ≥45 years), domicile (Central Jakarta, West Jakarta, North Jakarta, East Jakarta, South Jakarta, Kepulauan Seribu), occupation (public, health worker, government staff), week of PCR test, and history of previous COVID-19 infection (>3 months before the index PCR test)

^a^ Range time since last dose 2 is 14–90 days before the index PCR test

^b^ History of previous COVID-19 infection (>3 months before the index PCR test)

^c^Time since last booster is 7–90 days

95%CI: 95% Confidence Interval; OR: Odds ratio; Vax+ : Vaccinated; Vax-: Unvaccinated; VE: Vaccine effectiveness

**Table I. Indonesia Ministry of Health’s recommendation of booster dosage regimen in January 2022**

| **Vaccine type** | **Dosage** |
| --- | --- |
| For individuals who received the CoronaVac (Sinovac) primary dose | |
| ChAdOx1 nCoV-19 (AstraZeneca) | Half dose (0.25 ml) |
| BNT162b2 (Pfizer) | Half dose (0.15 ml) |
| For individuals who received the ChAdOx1 nCoV-19 (AstraZeneca) primary dose | |
| mRNA-1273 (Moderna) | Half dose (0.25 ml) |
| BNT162b2 (Pfizer) | Half dose (0.15 ml) |
